# “Whatever *Did* Ever Happen to the Likely Lads”? Social Change, Mobility, Class, and Identity in the UK 1969–2019.

**DOI:** 10.3389/fsoc.2020.541490

**Published:** 2020-12-08

**Authors:** Jon Warren

**Affiliations:** Department of Sociology, Durham University, Durham, United Kingdom

**Keywords:** class, de-industrialization, identity, social mobility, after industry, precarity

## Abstract

This paper reflects upon issues of class and identity in the UK over the last 50 years. 1969 is a useful starting point, economically and technologically it can be regarded as the high tide of the vision of a Britain remade in what the then Prime Minister Harold Wilson had termed “the white heat of technology.” That technology had produced Concord the world's first supersonic airliner which made its debut that year. This successful Anglo French collaboration also showed how Britain was rapidly moving into a different relationship with Europe a process that culminated in the UK's entry into the EEC or the “common market” as it was more usually referred to on January 1st 1973. Sociologically, it marked the publication of Goldthorpe et al. Affluent worker studies, which examined the idea that increasing affluence was breaking down established class structures, roles and attitudes. This debate about whether the changing nature of work brought with it a fundamental change in class structure and identity has been going on ever since. It has subtly changed and this is to be expected, 50 years is a long time. However, it is generally accepted that the change to economic and social policy that had followed the second world, social mobility was increasingly evident by the late sixties and continued into the seventies. Over the years issues of not only social class but de-industrialization, social mobility, regional decline, globalization, and its impact on policy have been added to the mix. Furthermore, “Brexit” is part of this debate and partly a product of it, however this paper doesn't have the space required to examine this. The title of this paper relates to Dick Clement and Ian La Frenais sitcom “Whatever happened to the Likely Lads” (WHTTLL) which was first broadcast by the BBC in 1973. It focusses on the relationship between 2 young men in their late 20's Bob and Terry. They come from the same place, have similar backgrounds and have been friends since childhood. But their lives are now at a crossroads. Whilst Bob is attempting to grasp new opportunities, Terry is skeptical and to some degree baffled by the change going on around him. The future of class in an era of social mobility also raised questions about aspiration and identity. These are questions which are now less prominent within discourse on class. The debate around class today is arguably less concerned with the mainstream and has become focussed on extremes looking at either poverty, criminality, dispossession or sometimes the lifestyles of a superrich elite. In summary then this paper will look at the changing ideas, and narratives that have surrounded social class and social mobility over the past half century within the context of the UK, it will do that by reflecting on my own personal biography and the themes raised in “Whatever happened to the Likely Lads” (WHTTLL).

## Background

### Biographies, People, Places, and Times

I am interested in biography, but not just in the conventional sense that is generally concerned with giving accounts of the lives of individuals (usually those who have become famous or notable for some reason). My interest is wider than that, I am not just interested in the biographies of people, but of the places and also the times that they inhabit. I make no claim that this is in any way novel. I see it as an exploration within the spaces between the three key intersections that Wright-Mills outlined in the Sociological imagination (Wright Mills, 1959). Namely those between Private problems and Public issues, the Individual and the Social and the interplay between Biography and History.

Mills saw all of these areas as important but it can be argued that he saw the relationship between Biography and History as particularly crucial, declaring that;

“Neither the life of an individual nor the history of a society can be understood without understanding both” (1959, p. 5).

This paper then is an exercise in exploring biographies and history. It has certainly been prompted by my own biography as during the course of writing this paper I celebrated my 50th Birthday.

I was born at the very end of the 1960's and as such my lifetime began when Britain was at its most highly industrialized, as Fothergill and Beatty have shown employment in 1966, just prior to my birth, employment in manufacturing and primary industries like coal reached a peak with 8.9 million workers employed in them. Today they account for just 2.9 million in other words my life span has coincided with the UK's rapid de-industrialization.

“Back in 1966, when manufacturing employment peaked, 8.9 million worked in manufacturing and a further 500,000 in the coal industry. This compares with just 2.9 million employed in manufacturing in 2016, and none at all in the coal industry” (Beatty and Fothergill, 2016, p. 4).

I have reflected upon my own biography and family history increasingly in the last few years, I expect this is partly a function of aging, but I have also become increasingly aware that the opportunities that were available to my parents and to me, are no longer available to my son and his contemporaries in the same way. The history of the last 50 years then has been about not only the de-industrialization of British society but also the narrowing of structures of opportunities. This is not to say that opportunities for social mobility have disappeared, however the risk and cost of taking up such opportunities has been increasingly shifted onto individuals whereas they were previously promoted and paid for by the state.

Perhaps it would be helpful to elaborate. My parents were born in Battersea, South London in the years immediately before the Second World War. Both came from typical working class backgrounds. My paternal grandfather was employed in the family trade, he was plumber and gasfitter, my paternal grandmother had been a housemaid when she first left school but then worked as a children's nanny. On my mother's side, her father had died young at the age of 41, he had been sailor in the Navy during WW1 before going to work for the Post Office. My maternal grandmother was left with two young daughters after his death in a world before the welfare state. The Post Office employed her as a cook where she spent the rest of her working life, although before getting married she had worked as a bookkeeper. Both families lived in private rented accommodation. Neither of my parents were evacuated during the London Blitz (by this I mean they were not sent away to live with strangers as so many were). But both were taken away by parents to locations outside of London at certain points in the war, my father to Lancashire in 1941, and my mother to stay with relatives in Scotland in 1944, at the height of the V1 and V2 attacks.

My parents benefited hugely from the coming of the welfare state, particularly in terms of education. My mother attended the local secondary modern before going on to take “O level” exams at Regent Street Polytechnic. My father passed the 11 plus and went to the local grammar school, passed his “O levels” began studying for “A levels” and had hopes of perhaps going to University. However, his father's early death meant that he had to abandon his studies and go out to work. It is perhaps this availability of work due to the economic policy at the heart of the post-war consensus which allowed the dramatic social mobility which followed in the decades immediately after 1945. This I think is often forgotten, or at least taken for granted, instead we prefer to talk about the social policies, education, the NHS, the huge expansion of social housing, the benefits system. But it needs to be remembered that this was all built upon a Keynesian economic strategy and a commitment to full employment. My parents often remarked that it in the fifties it was possible to “leave a job on Friday and get another on Monday.” Whilst I have always been a little skeptical about the literal truth of this statement, I have no reason to doubt that jobs were not scarce and easy to come by. So by their early 20's my parents both had good white collar jobs, my mother in the insurance industry, and my father in engineering working in sales. By 1966 my father had a company car (he told me he had to pass a company driving test before he was allowed to use it). They had also moved out of inner London to suburbia. They bought a flat in North Cheam, this was made possible by a mortgage scheme provided by the then London County Council. By the time of my birth in late 1969 they had moved again to a 1930's semi-detached house still in North Cheam.

What did this all mean? Well it is difficult to be definitive, but it certainly meant that I would have a very different life with different opportunities to that which my parents had. It is the dramatic rise in social mobility that my parents' generation experienced and it consequences that is the backdrop to this paper and the main theme running through it. In other words what happened was both intragenerational mobility (the extent to which people can climb the earnings ladder within their own lifetime) and consequently escaping intergenerational mobility (the extent to which your parents determine your life chances) themselves and consolidating it for their children. Or more simply, my sister and I had more opportunities than our parents.

What happens to people is shaped greatly by their surroundings. I have written fairly extensively about how places possess their own biographies (Warren, 2011, 2017; Warren and Garthwaite, 2014), stressing the need for researchers and policy makers to understand local history, culture and practices, if an meaningful sense of place is to emerge and that this essential if the lives of individuals are to be understood and social and economic issues tackled. This paper is not so much concerned with place (although it certainly shouldn't be discounted), rather while place is of course in the background it is time which is more significant.

Time also seems to have become increasingly important as the period of unprecedented economic security and social progress from 1945 to 1979 that British commentators termed “the post-war consensus. This unwritten agreement between the political parties which accepted a mixed economy, full employment, and the welfare state had underpinned the type of social mobility which my parents and many like them experienced.

However, this era is becoming an increasingly distant memory this was an exceptional period and is now best regarded as such, although during the immediate post war and until the late 1970's this was thought to be the new normal. How we might return to that time and whether a return is desirable or even possible can be argued to have been the central debate in British Politics between 1979 and 2010. It has also been part of a wider debate which incorporates social scientists, for example, British social policy analysis has concerned itself to a very great extent with the progressive dismantling and sometimes partial reconstruction of the British Welfare State, whilst also attempting to predict what the social consequences of this might be.

What is certain is that the type of opportunities available for many have changed, and that we now live in a society which is far more unequal than it was in 1969 with less opportunities for social mobility of the kind that my parent's generation experienced. This change has had different consequences for those from particular backgrounds and undoubtedly location has also been an important factor.

People like myself born at the end of the sixties benefited as our parent's generation had already experienced social mobility, they had white-collar jobs and aspired for their children to achieve further and consolidate their position. In short they had done the hard yards it was now up to us to make the most of those gains. Although It can also be argued that social improvement had increased across the board between 1945 and 1969 but it was only those in certain industries and parts of the country who were able to withstand the economic pressure of the 1970's after the 1973 Oil crisis, the coming of Thatcherism and the economic recession of the early 1980's, who were able to hang onto these gains. Others of course were not so fortunate.

This was particularly the case for young working class men in places such as Teesside, which suffered a sharp reversal of its fortunes and went from having the highest GVA (Gross Value Added: Regional gross value added is the value generated by any unit engaged in the production of goods and services), apart from Aberdeen or Central London in 1971, to parts of Middlesbrough having rates of male unemployment in excess of 50% by the mid 1980's. David Byrne writing in 1995 summed up a key aspect of this change.

“In 1971 most school leavers in Middlesbrough had no formal qualifications but had access to an employment system in which many well-paid jobs did not require such qualifications. Now things are very different” (Byrne, 1995, p. 112).

What Byrne is referring to is how the decline of the staple industries of Steel, Chemicals, and Engineering in a place like Teesside had also meant the dramatic contraction of training opportunities for school leavers which existed in the form of industrial apprenticeships and training schemes. These had provided school leavers with qualifications and acted as the gateways to secure well-paid employment. Their value became more apparent as employment declined on Teesside as many trades had transferable skills, for example instrument artificers who are always in demand in the global petroleum and chemical industries.

What this highlights is that for many working class families the general rise in living standards that the post 1945 social and economic consensus had delivered, were not as permanent as they initially may have appeared. Furthermore, those who had by a combination of these broad social improvements combined with their own efforts to “better themselves,” had undoubtedly changed their situation. They also expected that the kind of social mobility they had experienced would also be available to their own children and their grandchildren after them. Such expectation then can be argued to be a product of a particular time. However, the reality was that the sustainability of that was strongly connected to the places they lived and worked in.

### Whatever Happened to the Likely Lads?–1973–1974

Dick Clement and Ian La Frenais' 1973–1974 TV series is very much a product of its time, I would argue that it captures ideas and anxieties about “embourgoisement” and the disappearance of what for want of a better term “traditional” working class life. This of course was one of, if not the central pre-occupation of British sociology in the sixties and seventies with debate emanating from the affluent worker studies carried out by Goldthorpe et al. and published in 1968 and 1969. This in turn fed debates about the nature of social class, what it was made of, what it wasn't made of, how to recognize it and how to measure it. Crompton writing in 2010 sums it up very well.

“the linkages between concrete classes and the structure of employment were brought closer together, sociologically speaking, as a consequence of an influential corpus of research and writing in Britain from the late 1950s onwards, including Lockwood (1958), Dahrendorf (1959), and Goldthorpe and Lockwood (1963). Goldthorpe and Lockwood's (1963) article gave notice of a major empirical project, on the ‘affluent worker’ (see Goldthorpe et al., 1968, 1969, 1970). The ‘affluent worker’ research was a case study of Luton which included three major manufacturing organizations (Vauxhall, Skefco, and Laporte). The researchers examined comparatively a range of manual occupations—skilled, routine assembly, chemical process work as well as ‘white-collar’ employees (the study focused on male workers only). One of the major objectives of the research was to examine the thesis of ‘embourgeoisement’, or ‘the worker turning middle class”' (Crompton, 2010, p. 12).

It is very clear within WHTTLL that the writers conceptualization of social class is a highly complex one, it is not just about work, money, politics, or heritage it is instead about all of these things and more. Place, attitude, clothes, leisure choices are all thrown into the mix. Its message is that class cannot simply be boiled down to one of these factors (This is something that social scientists have sometimes found difficult to grasp and deal with, this is a theme that we shall return to later). But the series whilst it explores class routinely it doesn't at any point get bogged down by the issue and it provides much of the comedy.

I was too young to see the series when it was first screened, however it has been extensively repeated on terrestrial and satellite TV on regular basis ever since the early seventies. I believe that its enduring appeal is because many of the themes it explores about change, place, class, tradition, and aspiration are still very much with us today. I think that it appealed to my parents as they saw much of their own situation being presented back to them. Many of my generation saw much of our parents in the characters in the show in my case Mum and Dad were recognizably Bob and Thelma Ferris.

The series also became of more interest to me as it increasingly intersected with my own personal biography. I came to live in the North East in the late 1980's to study Social Sciences at what was then Sunderland Polytechnic. Apart from a short time overseas in the early 1990's I have lived in the region ever since settling here and raising a family. The rise and fall of the North East and its industries has also over the years become my chosen subject of study.

I arrived when the region's traditional industries, coal mining, iron, and steel making and ship building were in their closing stages whilst others which are now central to the region's economy, like automotive manufacture were just beginning (Nissan opened their plant at Washington in 1985). This tension between the region's past and its future has been a theme I have encountered again and again. What was and what might be is also at the heart of “Whatever happened to the Likely Lads,” hence it has been a text that I have returned to many times over the years.

I am very conscious that a British TV situation comedy made in the early 1970's might not be the most accessible text for those reading this journal (although the series can often be found on streaming sites such as YouTube, or Dailymotion links can also be found in the references section for those of you that wish to do so). Consequently there is a need to introduce its main protagonists, Bob and Terry.

The trajectories of the two central characters Bob Ferris and Terry Collier can be argued to offer us a way to track ideas about class and identity and the region through fictional but essentially recognizable characters. How they were formulated and how enduring they have been, is in itself worthy of discussion. Basically the series relates the adventures of and the relationship between these two former school friends and workmates who meet again after losing touch for several years.

The stories revolve around their aspirations (or in Terry's case the lack of them) and their common history. They were born at the end of the second world war went to school together and then went to work in the same factory and both were fairly typical young skilled industrial workers. This was how they appeared in the earlier series (1964–1966) ‘The Likely Lads.’ The original series ends as Bob decides to join the Army to “see the world” and “do something different” (It is worth noting that this was a fairly common thing, joining the armed forces offered a way to move away from home for young men, although in the North East a far more common way of getting away from the region was by joining the Merchant Navy). However, Terry although initially scornful of Bob's decision to leave him, his home and his job behind misses his friend and he decides to join him in the army. However, as Terry arrives to begin his basic training he is dismayed to see Bob coming the other way. Bob has been discharged on medical grounds for having “flat feet” and is returning home leaving his best friend in the care of HM Armed forces. Thus, the parallel paths which their lives had followed up to this point diverge forever.

By the time we meet Bob and Terry again in the 1973 series their circumstances are very different. Bob has moved on and whilst he not ashamed of his working class background, aspires to “better himself” in terms of both his income and lifestyle. Bob has left the factory that he and Terry joined straight from school, and we learn that he has gained more qualifications partly through a correspondence course and through “night school” (this alongside other initiatives such as “day release” schemes, operated by large employers which allowed workers to attend Further and Higher Education courses during the working week were an important part of post war mobility). Bob is working as a site surveyor for a construction company and has essentially become middle management, it must also be said that being engaged to the bosses daughter hasn't harmed his prospects either. So Bob's working life has changed dramatically in the 5 years that we are told have elapsed since his return from his brief time in the army and Terry's departure.

It is not only his work situation that has changed. Bob is about to get married, he is buying his own house on the “Elm Lodge Housing Estate” a new build estate on the edge of the city, buying his own car, and he goes on foreign holidays. Whilst such things are commonplace now they were not in the early 1970s. Bob also feels that he has a new social status too. In other words the change of his employment status has led to a different social status. Bob and his fiancé Thelma who he has known since junior (primary) school are aspirational and anxious to leave their past behind, this means living a different sort of lifestyle with different friends, different activities, and with different habits. The past is just that, the past, something to be fondly remembered, but something that Bob and Thelma are glad that they have left behind. However, this is less of a departure for Thelma as her father is a self-made man who runs his own company and she herself works as an assistant in a local library. It is the friction that this supposed change of social status or the aspiration to change social status that provides the space or situation for much of the comedy to unfold.

The past then is given both form and a dissenting voice by Terry. Terry has now returned home from the army and he's shocked to find how things have changed. Terry struggles to grasp and accept how his home town has changed, but he is even more alarmed by the change in his friend. As the series progresses, it becomes clear that despite their apparently diverging lifestyles, their common past binds them together whether they like it or not.

Put another way the characters can be seen as personifying what Williams (1973) termed the “emergent” and “residual working” class. This process remakes and reproduces the relations of class in new and novel forms. Class relations do not fundamentally alter but their form changes.

“By residual I mean that some experiences, meanings and values which cannot be verified or cannot be expressed in the terms of the dominant culture are nevertheless lived and practiced on the basis of the residue cultural as well as social of some previous social formation. By “emergent” I mean first that new meanings and values, new practices, new significances, and experiences are continually being created. But there is a much earlier attempt to incorporate them because they are part-and not yet part of effective contemporary practice” (Williams, 1973, p. 10–11).

This interplay continues throughout the series, I find this particularly fascinating as the characters can also be read as shorthand for the North East region too which has an extremely strong “residual culture.” This strong heritage derived from Coal, Iron and Steel, Heavy Engineering and Shipbuilding, which established the North East as the world's first industrial region in the nineteenth century still plays an important part in the regions life and identity to this day. However, this incredibly powerful complex of what Byrne has termed “carboniferous capitalism” (Byrne, 2005a) which the regions power and wealth was founded upon had been in decline since the early twentieth century as other parts of the world industrialized and caught up. It's highpoint can be argued to be 1913, which was the year that the great Northern Coalfield which covered County Durham, Northumberland and what is now Tyne and Wear achieved its highest ever production figures. The last deep mine in the North East closed in 1993, but mining heritage is celebrated by the region. As Tomaney points out in this collection the annual Durham Miner's Gala or “Big Meeting” which is held every July and attracts huge crowds in what has been described as the “biggest celebration of working class culture in Europe.” It can be argued then that the search for a new identity for the region has been going on for a very long time, and that finding and nurturing an emergent culture has been an uphill struggle.

At the time of WHTLL in the early 1970's the region still had its traditional industries (although they were well into their long and often painful decline). Shipbuilding continued on the Tyne, Wear and the Tees. Iron and Steel still had a major presence at Consett and on Teesside. Coal was still a major source of employment but many of the region's inland collieries had closed in the 1960's and workers transferred to the larger coastal pits or to other parts of the country. It was the need to consider what “life after coal” looked like that led to the 1963 Hailsham report on the North East (HMSO, 1963). It recommended major infrastructural improvements many of which the region had wanted for years such as the final construction of the Vehicular Tyne Tunnel (something agreed upon as long ago as 1937). Roads were upgraded and new town developments approved. The report envisaged the region as being a center of advanced manufacturing. This mirrored (in fact it predated) Wilson's vision of a Britain “forged in the white heat of technology “and it also bolstered the dominant narrative in local and regional politics which was one of “modernization.” At the heart of this modernization agenda was the deliberate fracturing of the regions past in order to recreate the place a new, for those leading this project, this meant ensuring that the region could not return to the poverty and unemployment it had been blighted by in the 1930's. This political project was personified in the shape of Thomas Dan Smith (1915–1993). T Dan Smith rose to prominence in the 1950's and 60's as leader of Newcastle City Council. Always a controversial character Dan Smith was loved by some to whom he was “Mr Newcastle” and loathed by others to who he was “the mouth of the Tyne.” Smith was intent on large scale change, he initiated ambitious modern building schemes and demolished large swathes of Victorian Newcastle in a bid to create the “Brasilia of the North.” Newcastle and many other parts of the city were certainly transformed by the revolution that “Modernization” brought. Dan Smith's political career ended in disgrace and imprisonment after his connections to a number of corrupt transactions involving the developer John Poulson came to light in the early seventies. His legacy for good or ill remains within the fabric of Newcastle and the wider urban North East.

It is this changing landscape that Terry has returned to and in an early episode entitled “Moving on” Bob decides to take Terry for a Sunday morning drive around their old neighborhood. Terry is enthusiastic at first but Bob warns him.

Bob: “Things have changed.”

Bob drives Terry around the town, Terry is eager to see their old haunts from their teenage years but at every turn Bob informs him that the dance halls, coffee bars, and pubs he remembers have been demolished with new structures under construction or having already taken their place.

Terry is both shocked and disbelieving. When Bob reveals that the “Go-Go Rock Club” is now buried under a huge multi story car park Terry exclaims.

Terry: “the Go-Go! Gone!”

The duo retire to the pub to have a pint and talk things over. Terry is dismayed and Bob admits that there have been a lot of changes, but adds that;

Bob: “if you live here all the time you don't notice it so much, still it's a good thing progress, expansion, plenty of opportunities round here now you know”

Terry counters this with,

Terry: “You wouldn't think that if you went down the labor exchange!”

Terry also learns that it is not just his old social haunts that have disappeared. When Bob asks him what his plans for getting a job are he is non-committal, when pressed on this Terry says that if nothing turns up he can always,

Terry: “go back to Ellison's (the factory they had both previously worked at) old Darby always said to me “when you come out Terry your job will be open” I'll just go back to Ellison's!”

Bob however has news for Terry.

Bob: “there is a problem, they pulled it down 2 years ago!”

What is striking about this scene to me is that many of the places which feature in it as the “new” buildings have themselves been demolished in the last 10–15 years. In fact the multi story carpark referred to became famous in its own right. Designed by Owen Luder as part of the Trinity Square shopping center in Gateshead the car park was part of one of the definitive scenes from what is certainly *the* definitive North East film Get Carter (1971). Jack Carter takes revenge upon one of his brother's killers by throwing them to their death from the top level. Featuring in the film led to the building achieving cult status. However, in spite of this this and attempts to get it listed building status, the “Get Carter” car park was demolished in 2010.

Bob and Terry's reflections on the changing landscape around them continue later in the episode as they join Terry's sister Audrey and her family for lunch.

Audrey “Still it's a good thing progress, expansion isn't it? There's a lot of opportunities round here now.”

Ernie: “Yes they say that by 1988 this will be one of the most exciting environments in the United Kingdom!”

Terry “is there anything still left standing in this town? One solitary pre 1967 brick left standing on top of another?”

It is clear that the absence of physical elements of his past unsettles Terry. However, Bob, Audrey, and Ernie insist that Terry will be ok as he has a trade to “fall back on.”

Ernie: “You're a qualified electrician you'll have no problem getting a job.”

The importance and status of “having a trade” should not be underestimated. Ian Robert's book “Craft, Class and Control” (Roberts, 1993) illustrates this very-well. Roberts explains how his decision to leave a craft apprenticeship as a pipefitter in the shipyards of Sunderland in the 1980's was met with incredulity by his family and friends. This went against all the received wisdom that an apprenticeship was to be prized as its completion was the best guarantee of employment available to working class young men in his community.

The question of employment returns to the series time and again, but Terry remains either unemployed or in unskilled work throughout, and certainly never works as an electrician.

The theme of work and its relationship to social class and social mobility is one which sociologists have made many attempts to unpick. The embourgoisement thesis, saw class as being intimately linked to work crudely making the transition from blue collar work to white collar work meant leaving the working class and becoming middle class (Undoubtedly many who made such a transition also saw this a being the case too).

Of course by the early Seventies this thesis was being challenged as the workplace again became the focus of industrial conflict. Whatever happened to the likely lads did after all first appear in the midst of the Miner's strikes of 1972 and 1973–4 which alongside the 1973 oil crisis reduced Britain to a 3 day week under the leadership of Conservative Prime Minister Edward Heath. Class conflict in the workplace had also become of interest to sociologists again (Beynon, 1973), saw the publication of Huw Beynon's seminal work on industrial relations in the car industry “Working for Ford.” The following year Braverman (1974) challenged the idea that a “change of collar” could bring about a change of class by arguing that white collar jobs were more accessible but were also increasingly routine and “proletarianised” as such it was not the type of work or the color of the collar that mattered but the relations within labor process itself.

This is not a debate which Bob and Terry really enter into, Bob has come to see work as vehicle away to earn the kind of money which will allow him to access a certain lifestyle, a lifestyle which will allow him to “get on.” Terry on the other hand sees work as a necessary evil, a means to an end, the way to earn enough money to pay for the good things in life which for him are rather more traditional, such as drinking, smoking, and gambling. Industrial relations are barely mentioned within the series which is fairly remarkable given the backdrop of industrial strife that was going on in the UK at the time. Instead we know that Bob is a site surveyor working for a construction company, owned by his fiancé's father (Bob of course insists that Mr. Chambers “does him “no favors” and that he has to “pull his weight”). Thelma works in a local library, this is important to note. Byrne (2018, p. 38) uses WHTTLL to make the point of how women's employment and the growth of dual income households where important components of the post-war transformation.

“What large scale female waged work also led to was a real growth in household incomes and of general household prosperity. So for example in the UK the mass growth of owner occupation whilst not solely due to the income levels of dual-income households was a crucial transformer of lived experience” (Byrne, 2018, p. 38).

This again mirrors my parents experience in the 1960's with a dual income being essential for them getting an initial foot on the property ladder.

Finally, Terry is a happily unemployed electrician. Occupation then, with the series is a signifier of social status but just one of several which indicate how far people have “got on.”

### Getting On ……

Within “Whatever happened to the Likely Lads” (WHTTLL) there is a wider discussion around the idea of “Getting on” which frames most to Bob and Terry's discussions about employment. In the second episode of the series Bob asks Terry what he is going to do now he is home and out of the Army, offering the suggestion that,

Bob “it's time to forge ahead and build a future!”

Terry: “Some of us have been able to forge ahead and build a future, while some of us have been serving Queen and Country.”

This theme reoccurs throughout the series Terry repeatedly tries to make Bob feel guilty/responsible for his time in the army and therefore his failure to “get on” (Of course in reality Terry could always have bought himself out of the army).

Again we see Bob looking toward an emergent future whilst Terry prefers to view the future as a distant almost threatening prospect from the relative safety of the past. Terry is both dismissive and somewhat jealous of Bob's success.

Terry: “You've obviously done alright for yourself, mortgage, and a car and a few premium bonds have you?

However, his compliments are often back-handed ones. Series one of WHTTLL is built around the run up to Bob's marriage to Thelma. They have bought a new build house on what is referred to as the “Elm Lodge housing estate” but are yet to move in (remember it's still the early 1970's and living together pre marriage is still an issue especially for the “respectable” working class). Instead Bob is spending time at the house decorating and preparing for when he and Thelma move in. Terry visits Bob at the house.

Terry to Bob “Is this your house?”

Bob proudly and enthusiastically begins to tell Terry all about the house and its specifications he concludes by saying,

Bob: “Yes, we haven't got a name for it yet.”

Terry: “Well you'll need one to find it again!”

Bob of course protests that not all the houses on the estate are the same, but his friend's criticism of his new home annoys him. Terry compounds this by, giving the following generalized critique of Bob's new home, lifestyle, and situation.

Terry: “There's something so depressing about these estates, it's the thought of you all getting up at the same time, eating the same kind of low calorie breakfast cereal, all coming home at the same time at 6.30 switching on the same programme at the same time and having it off the same two nights of the week!”

By this time Bob has had enough and he counters Terry's provocation with a defense of his aspirations.

Bob: “Well what's wrong with being the same as everyone else? What's wrong with wanting to make a bit of modest progress? I've worked hard for the last 5 years and this is what I've got to show for it. This and the car. It might not mean much to the Burtons but it means a hell of a lot to me! And what's so special about your lifestyle? You're homeless, jobless, car less, and single. What does that amount to? Terry Collier, bachelor pedestrian!”

Again, Bob talks about “progress” by which he means social mobility, and whilst Terry is at best skeptical and at least mocking of this, but he doesn't have an alternative vision to offer.

It is also worth noting that homeownership is central to Bob's aspirations. This key of “getting on” was of course to become increasingly important to UK politics over the next decade as the promise of a “property owning democracy” proved central to the election of the Conservatives in 1979 and the Thatcherite project. Politics are never really openly discussed in WHTTLL but it is clear that Bob and Thelma would be key targets for the Conservatives.

The way then that Clement and La Frenais present issues of class, social mobility, and social aspiration in the series has far more in common with the ideas of Pierre Bordieu, than other theorists. This is essential as it makes Bob and Thelma much more human and likable. Without this Bob and Thelma would just be examples of embourgoisement. Instead the writers explore the contradictions in the characters and the problems with the assumption that Bob has become middle class by getting a white collar job and buying a house and a car.

Bourdieu argued that individuals and their communities are molded by the culture which surrounds them and they develop a particular mode of living. Bourdieu used the related ideas of capital, habitus and field, in order to explain this.

Bourdieu argues that capital consists of several elements; economic capital i.e., material resources, assets, and earning ability. Social capital, circles of friends, memberships of groups, and social networks. Cultural capital, individuals' skills, knowledge, life experience, taste, and manners that are acquired by being part of a particular social class. Cultural capital is a powerful mechanism which leads to social exclusion and inequality as some forms of cultural capital are seen as much more valuable than others. So for Bourdieu the more capital one has, the more powerful a position the individual occupies in social life. Habitus is the ingrained habits, skills, and dispositions that we possess due to our life experiences, it is the manifestation of capital which we effectively carry around with us. It is our habitus allows us to successfully navigate social environments, however if those environments are beyond our experience this does not work i.e., it is depended upon context. Bourdieu argued that habitus was often taken for granted and mistaken for being natural rather than cultural. The final element is what Bourdieu called “Field.” The social world consists of a multiple fields which overlap such as work places, institutions such as school, family, education, religion, and practices like art law and politics i.e., the spaces within which social life occurs and make up society. Bourdieu contended that each field had its own rules and practices which he termed “Doxa” (Longhofer and Winchester, 2013).

However, what is uncertain is how capital and the related habitus formed in areas dominated by industrial work and the social relations which underpinned them endure or change as the social and economic fabric changes? Is the old habitus the basis of a new modified habitus? If so how significant is this, and what happens when the goalposts are shifted radically? Does this mean the past has more to do with the present than we might like to think? Or is the habitus of the past an obstacle to be overcome? All of these questions can be found within WHTTLL.

For people in Bob and Thelma's position like my own parents in the early nineteen seventies it is fairly clear that their context and surrounding, the time and place within which they live combined with their own personal agency meant that acquiring economic capital was not the prime issue, instead the question of accumulating social, and cultural capital became much more important. This process is far more difficult and throughout the series scenarios within which Bob tries to accumulate social and cultural capital whilst being haunted by his habitus in the form of Terry are a recurrent theme.

These elements come to the fore in the eighth episode of series one “Guess who's coming to dinner.” Bob asks Terry to come with him and Thelma to dinner at their “new” friends Alan and Brenda's house. Initially Terry is hostile to the idea and refuses. Bob teases Terry likening him to the cartoon character “Andy Capp” (a well-known cartoon which first appeared in the *Daily Mirror* in 1957, Andy is a northern working class caricature, workshy and found frequently in the pub or the betting shop, hence the comparison with Terry). Initially Terry decides to treat this as a compliment and attempts to turn the conversation back onto what he sees as Bob's middle class pretensions.

Terry: “let me tell you something, I *love* Andy Capp! I'm proud of my home and my class. Just because you're flirting with the lower lower middle middle, just because you've got yourself an office job and fiancé who lives on a mock Tudor estate with a monkey tree!”

He continues in the same vein.

Terry: “I don't want to meet any of your new friends. I bet they all belong to the rugby club, the tennis club or the squash club!”

Bob simply replies,

“We all have a wide range of leisure activities if that's what you mean”

For Terry leisure activities are indicative of social class, i.e., they are for him indicators of social and cultural capital. He attempts to hammer this point home to Bob who of course recognizes this too but sees things rather differently.

Terry: “You'll stop going to football soon, because Saturday afternoon will be *golf!”*

Bob decides to leave Terry to it.

Bob: “if you want to sit there behind your class barrier, fine.”

However, Terry eventually agrees to accompany Bob and Thelma to dinner at Alan and Brenda's the following weekend.

Saturday arrives and Bob and Thelma with Terry in tow arrive at Alan and Brenda's. It is quickly established that whilst Alan is an immigrant from “the deep south” (in fact Surrey) Brenda had attended Park Junior School along with Bob, Thelma and Terry. This provides the set up for the rest of the episode which continues to stress class, aspiration, social capital and snobbery.

Brenda makes it clear from the outset that she doesn't want to discuss the past.

Brenda “I'd like to think that we have come a long way from Park Juniors.”

Terry however has no other template to reference except the past and responds,

Terry: “Well you certainly have Brenda the last time I knew you, you were living above your dad's chip shop near the glue factory.”

Brenda is embarrassed and annoyed, it also becomes clear that this is an aspect of her past that Alan is unaware of.

Alan “Did your father have a fish and chip shop dear?”

Before Brenda can answer Terry steams in with further details much to Brenda's embarrassment.

Terry: “Yes he did, a good ‘un and all, the Silver Grid, big helpings and free batter!”

Of course this is in Terry's eyes a compliment, Brenda struggles to contain herself and excuses herself by saying she has to go and see to the dinner. Terry fans the flames further by commenting,

Terry: “Are we having rock salmon and chips for old time's sake?”

With Alan and Brenda absent in the kitchen Bob feels compelled to ask Terry to avoid the subject of Brenda's past. His reasoning is,

Bob: “There's nothing wrong with someone who gets a bit of money and a nice house wanting to forget their past. If you must go down memory lane leave off her dad and the chip shop.”

Dinner passes uneventfully until the subject of the past is brought up again, not by Terry this time but Bob who offers some food for thought.

Bob: “Here we all are having a sophisticated supper party with “Tia Maria “and wafer thin mints, and the last time we sat down to eat together was school dinners at Park Juniors.”

A discussion ensues between Bob, Thelma, Terry and Brenda about school dinners whilst Alan looks on with interest. Brenda cannot see why the others are still interested in a past she would rather forget.

Brenda:” that's irrelevant now we are all different people!”

Bob, however begs to differ about this.

Bob: “Not *necessarily*, not *necessarily*, we might be a bit older we might have a few more possessions now but we are still basically the same people.”

Bob and Thelma are happy to discuss the past. For them it is something that they retain but feel that they have moved away from. Unlike Brenda who would rather hide her past, or Terry whose only reference point and guide to action is the past. Put another way this issue is not just about the past but their relationship with their habitus in Bourdieu's terms. It also provides the backdrop for the climax of the episode when matters come to a head. Brenda can finally take no more of the tales of Park Junior School and she turns her anger toward Terry.

Brenda: “Part of your reluctance to leave the past Terry Collier is that you have very little to look forward to in the future, and your present hasn't much to offer beyond the pub and the billiard hall. Most of us have improved ourselves and developed as people. But you, you're an embarrassment to your family and an embarrassment to your friends!”

Bob swiftly leaps to Terry's defense.

Bob: “Now wait a minute! He doesn't embarrass me! He might be coarse and he might be vulgar. He might be crude and bit rough at the edges, and all right he might have eaten the wrong end of his asparagus, but I'll tell you one thing, he's down to earth and he's honest.”

This is not all that surprising given Bob and Terry's common history and long friendship. What does come as a surprise is that Thelma also comes to Terry's defense, and turns things back onto Brenda.

Thelma: “I think Bob's right. Terry is honest, he's got no pretensions, *he* would never deny he lived above a chip shop! You're an enormous snob Brenda and you always have been!”

Brenda is shocked and challenges Thelma, who is happy to carry on criticizing Brenda accusing her of flaunting her new found affluence and possessions.

Thelma: “When you watch people admiring your fabrics and praising your carpets and envying your precious fondue set, you wet your knickers!”

Unsurprisingly this brings the evening to an abrupt close!

Of course these kind of comedic takes on class, social mobility, and aspiration was an ongoing theme in comedy and comedy drama in the 1970's. Mike Leigh's Abbigail's Party celebrated (Leigh, 1977) play explores very similar themes in a similar situation to “Guess who's coming to dinner,” but it does so in much greater depth. However, can it be said that these issues are specific to a particular time? Britain has changed a huge amount since the 1970's do these anxieties about class status mobility and escaping from circumstances and habitus have any relevance today?

It can be argued that they do. Katy McEwan whose blog post “Drinking Carling out of Stella glasses” (McEwan, 2016) addressed this question as part of her study of Ingleby Barwick.

Ingleby Barwick is today's Teesside equivalent of the “Elm lodge housing estate” and Bob and Thelma Ferris would probably be found living on, or aspiring to move to Ingleby Barwick. As part of my research about Teesside and the legacy of the industrial past I interviewed Katy about her work.

Katy explained that the people she spoke to were aspirational and from working class backgrounds, but in a particular way, they were keen to transform themselves into something else by their physical relocation to Ingleby Barwick. Moving there was about leaving the past behind this physical transition they saw as bringing a new status. Katy told me what she thought lay behind this:

“The purpose is to not be where you have come from.”

This involved a change of location and also one of mind-set:

“It's about becoming different people, about no longer being one of them.”

In this case “them” means the community you were part of previously so it is both a physical and cultural departure (McEwan in Warren, 2018, p. 205).

It would seem then that very little has changed in the intervening 40 years, what is being described is a very similar process that Clement and La Frenais dramatized back in Clement and La Frenais (1973). But of course things are very different too.

### What Did Ever Happen to the Likely Lads Then?

The situation which is so well-outlined in WHTTLL is still with us in many ways. But the intersection of personal biographies and the economic landscape is not the same, times have changed. In a world where class is still important but operating in a post-industrial context things are a good deal more uncertain than they were in 1973.

The question of whatever became of Bob Ferris and Terry Collier has been speculated about over the years, with the question re-emerging on a regular basis. Alas plans for any kind of update or follow up on the characters situations were never to be after the actors Rodney Bewes and James Bolam who played Bob and Terry fell out during the filming of the likely lads feature film in 1976 and never spoke again. Any possibility of a reconciliation and revival was finally ended with Rodney Bewes death in 2017.

Clement and La Frenais have responded to the question of what might have become of the characters their replies have been plausible but ambiguous along the lines that Bob Ferris started his own business and went bankrupt in the 1980's whilst Terry Collier became a millionaire scrap metal dealer…or got a vast compensation pay out after being involved in an accident.

In a 2019 La Frenais in an interview with the Times explained that,

“Through no fault of his own but circumstances, in post-Thatcher Britain... [Bob's] business ran into financial difficulties so it was a real struggle. After all those years of wanting to upgrade, Bob is forced to downgrade and retire”. Whereas, Terry was involved in a serious car crash that ended up being lucrative for him.

“Probably he drank a bit, he was probably coming back from a race meeting, and he scored an enormous amount of money.” (The Times 18/03/2019)^1^.

What exactly happened to Bob and Terry the characters is of course something we will never really be sure of. In these interviews I always get the impression that the writers are really just humoring those answering the question, Clement and La Frenais moved on from the Likely Lads over 40 years ago and a lot of water has flowed down the Tyne since then. However, It is interesting that Bob's story is very similar to the of Dennis Patterson who was one of the main characters in Clement and La Frenais highly successful 1980's series “Auf wiedersehen pet (Clement and La Frenais, 1983)”. In the series about unemployed building workers from the North East traveling to Germany to find work Dennis is introduced as having lost his own building firm to bankruptcy.

What is of more interest to a social scientist like myself is what happened to the “real” people, the parts of the population which Bob and Terry were meant to represent? Again, it is possible to construct possible life trajectories based upon the situations they found themselves in in 1973/74. As skilled workers both of them would have been courted by Thatcherism indeed successfully securing the votes of what were then known as C1 (skilled manual workers) was central to the Thatcherite project. This cohort are in many ways pivotal as they are the group which voted for the Conservatives under Margret Thatcher between 1979 and 1987 this period of course marked the end of the Post War consensus, and its replacement with neo liberal approaches to economic and social policy.

If I look at my parents fortunes in the 1980's they changed through that decade, it began well with my father having risen to be a senior project manager with a major engineering company. We moved from outer London further into the Home Counties with my parents taking out a far larger mortgage to finance the move. At the time such moves were encouraged by a government tax break provided by the Miras (Mortgage interest relief at source) scheme. However, the trajectory of upward mobility which my parents had been on all their adult life began to falter and stall for them by the mid-eighties. The recession of the early eighties hit engineering particularly hard and my dad was made redundant, fortunately he found work quickly but then moved through a succession of similar jobs. None however represented advancement on the career ladder or brought in a higher income. Interest rates also rose dramatically in the late 1980's and what had been a large but manageable mortgage became increasingly uncomfortable. This was not a disaster by any stretch but it was clearly to my father in particular a disappointment. So in many ways my father's experience was similar to the fate Clement and La Frenais described for Bob in the 1980's, not in terms of bankruptcy but certainly, he became increasingly disillusioned and disappointed.

My father was however heartened by the fact that I went off to do a degree in 1989, the first in my immediate family to do so, although he would not live to see me receive it. Its completion was only possible to the fact that I benefitted from the local authority paying my tuition fees and the fact that I received a maintenance grant to cover my living expenses. The gains then of my parents' generation coupled with parts of the state structure that had promoted social mobility allowed me to consolidate the gains that they had made.

It is worth asking whether these characters and their situations would still be of interest to social scientists. It can be argued that questions about class have increasingly become focussed on the margins and extremes. Rather than social class, social exclusion and the processes that cause it came to the fore. This is of course to a degree understandable given the changing economic background throughout the last fifty years. It can be argued that an interest in those marginalized by de- industrialization in times of rising affluence such as Smith (2005) book “On the margins of inclusion” rightly seeks to understand the processes that meant that many communities had been left behind whilst those around them were enjoying higher living standards than ever before. The 2008 financial crash and the ensuing rush toward neo-liberal austerity has rightly kept the spotlight upon those most marginalized by the draconian cuts in public spending and a remodeled and an increasingly punitive benefits system has meant that ethnographies of lives on the margins such as those by McKenzie (2015) and Garthwaite (2016) have rightly been at the center of sociological debates about class. Sitcom's changed to of course to reflect the times, they had different preoccupations and treated class very differently to WHTTLL. As I have already mentioned Clement and La Frenais' “Auf wiedersehen pet” reflected the reality of mass unemployment in the North East in the early 1980's. While “Shameless” (Abbot, 2004) which ran from 2004 to 2013 was based around life on a fictional Manchester “sink” estate. But Bob and Terry were never on the margins and this is what gave then their appeal to a great extent. Instead they have much more in common with what Byrne (2005b) has called the “missing middle” within his article he also cites the first time owner occupiers of Ingleby Barwick as being important but neglected by those of us trying to understand contemporary class.

Bob and Thelma Ferris are still very much with us then but in a modified form. The social mobility which had led to Bob and Thelma's move to the “Elm lodge housing estate” can be argued to have arisen as part of a collective social endeavor, the post war welfare state and its associated economic policies. The opportunities were made available and the likes of Bob were able to take them.

It can be argued that those who are buying homes at Ingleby Barwick studied by McEwan today are doing so for similar reasons but in different circumstances. Average house prices on Ingleby Barwick are relatively modest compared to other parts of the country, the property site Zoopla gave an average house price of £205,000 for the estate and £235,000 for detached properties^2^. However, it is unlikely that household mortgages for these properties equal 2.5 × annual income as would have been the case for Bob and Thelma. Also a dual household income is essential for today's Ingleby Barwick resident. It can also be argued that things are much more precarious. Yes it is much easier to obtain loans and credit, all will have one if not two cars financed by a personal credit plan (PCP) on the drive but they are much more likely to be employed insecurely. They are also likely to be carrying levels of debt that would have been incomprehensible to their contemporaries from 1973 in terms of both scale and type, for example student loans. This is a social mobility upon which the responsibility and risk is placed upon the shoulders of individual citizens. The big difference between the lives of those buying their first homes on Ingleby Barwick and the fictional residents of the Elm Lodge housing estate is that theirs is a much more precarious mobility which could be reversed very quickly as a consequence of higher interest rates or sudden job loss.

Precarity then it seems has become a fact of life, a new context within which both the “left behind” and the aspirational are forced to try and operate within and which shapes the biographies of individuals and the places which they live in. Due to this there is then a diminished ability for individuals to change their own circumstances due to the lack of a secure economic foundation upon which to build. Consequently, the social mobility which was possible for those from working class backgrounds in the post war decades has declined.

Social mobility is a critically important aspect of society. The post war consensus contained within it, an acceptance and understanding that if you don't give people opportunities then they can't take them. It was also accepted that this would be beneficial not just for the individuals who managed to “get on” but for society in general. Therefore, the state became an agent and sponsor of social mobility.

There has in recent times been attempts to ask the question “Whatever happened to social mobility?” The former Labor cabinet Minister Alan Milburn was appointed in 2010 by the Tory/Liberal Coalition Government to chair a Commission on Social Mobility which eventually reported in 2017 it reflected on the efforts made by governments of all political persuasions between 1997 and 2017 and conclude that,

“successive governments have failed to make social mobility the cornerstone of domestic policy. Over two decades efforts have waxed and waned. They been piecemeal rather than holistic. All too often they have relied on the tenacity and commitment of individual Ministers rather than being a core objective and shared mission for all Ministers, including Prime Ministers. The social mobility agenda has tended to be skewed toward children and the education system with too little emphasis on young adults and the labor market. There has been no overall long term plan for change” (Social Mobility Commission, 2017, p. 5).

I would argue that the reduction of opportunities has been largely due to a combination of neo liberal ideology which did not believe the state should sponsor social mobility directly, i.e., there has been no long term plan because there was no belief in planning and importantly industrial decline. The issue has been further complicated because the process of industrial decline has not been uniform with many regions of the country suffering disproportionately. As has already been stressed, the biographies of individuals are intertwined with the biographies of the places they inhabit. So the consequences for those living in the North East and other old industrial regions have been much greater than in other parts of the country. The North South divide may have been evident culturally in the early 1970's but it did not become an economic fact until the 1980's.

## Conclusion

In conclusion then it can be argued that there is an underlying, taken for granted assumption within WHTTLL that the social progress and mobility that had occurred in post war Britain was something that was permanent and irreversible. This new world of social possibilities was mirrored by a changing world in which the physical Victorian nineteenth world that had produced traditional class relations was being demolished and replaced with a new one based on modernization.

However, it also failed to see what the effect would be of abandoning a mixed economic model within which the government underwrote minimum levels of employment and income would be. Also how quickly seemingly permanent improvements could be reversed. But of course why should a sitcom consider such matters? They are there to entertain, but of course to be popular and successful they must represent lived experience to some extent. In the final episode of the second series of WHTTLL “The shape of things to come.” Terry's great Uncle Jacob dies, no one in the Collier family has a good word to say about him. Bob and Terry reminisce about him, but unsurprisingly the discussion turns back to the present and they resume their usual “residual” and “emergent” roles.

Terry: “I'm working class like he was and proud of it if that's what you mean!”

Bob: “So am I!”

Terry: “Get away! You used to be!

Bob: “I'm no less working class than you! We went to the same school, grew up on the same streets, and lived in the same drafty houses. But that was in my past, you still like to live like that. You like the old working class struggle against the odds. What you don't realize is that some of us have won the struggle, and it's nothing to be ashamed of!

Almost five decades on what looked like cynicism on Terry's part looks rather more measured and in hindsight it is Bob who can be accused of being naively optimistic.

So what has changed? The central question in WHTTLL was about the impact of economic and social change and the mobility it brought upon class status and relationships. Half a century on such concerns, whilst they are still present have faded into the background. Instead the central issues are about whether social mobility is still possible and if so on what basis? Savage (2011) in a report for the Resolution Foundation found that while intergenerational mobility (the extent to which your parents determine your life chances) has remained stubbornly steady, but intragenerational mobility (the extent to which people can climb the earnings ladder within their own lifetime) has shown some improvement.

“Our analysis shows that an individual's earnings at 40 were less closely tied to his or her earnings at 30 in the 2000s than in the 1990s, indicating an improvement in relative mobility. This challenges the generally held view informed by analyses of intergenerational income mobility that social mobility in Britain has stalled” (Savage, 2011, p. 24).

So it seems that if social mobility is possible and that Bob and Thelma Ferris's equivalents are out there any mobility they experience will be much more precarious and difficult to negotiate than it once appeared.

## Author Contributions

The author confirms being the sole contributor of this work and has approved it for publication.

## Conflict of Interest

The author declares that the research was conducted in the absence of any commercial or financial relationships that could be construed as a potential conflict of interest.

## Footnotes

^1^The Times. What did happen to the Likely Lads? Now we know (accessed March 18, 2019).

^2^Zoopla – House prices in Ingleby Barwick (06/03/2020). Available online at: https://www.zoopla.co.uk/house-prices/ingleby-barwick/.

## Specific Episodes of “Whatever Happened to the Likely Lads” and Streaming Links

WHTLL Series one episode 4-“Moving on” https://youtu.be/1S3wXNAeeLE

WHTLL Series one episode 2-“Home is the Hero” https://youtu.be/IJ1u-APoWPg

WHTLL Series one episode 8 -“Guess who's coming to Dinner?” https://youtu.be/G_KJZJ6HDMQ

WHTLL Series two episode 13 -“The shape of things to come.” https://youtu.be/Nwg7L3RHb48.
